# Effectiveness of an eye movement desensitization and reprocessing intervention for the prevention of post- traumatic symptoms in perinatal loss: a randomized pilot controlled trial

**DOI:** 10.3389/fpsyt.2025.1593306

**Published:** 2025-06-09

**Authors:** Bàrbara Sureda-Caldentey, Cristina Garcia-Gibert, Amparo Martínez, Yolanda Giménez, Xavier Segú, Aida Mallorquí, Estel Gelabert, Alba Roca-Lecumberri, Anna Torres-Giménez

**Affiliations:** ^1^ Unitat de Salut Mental Perinatal CLINIC-Barcelona, Hospital Clínic, Barcelona, Spain; ^2^ Psychiatry and Clinical Psychology Service, Hospital Clinic, Barcelona, Spain; ^3^ Department of Clinical Psychology and Psychobiology, University of Barcelona, Barcelona, Spain; ^4^ Sexual and Reproductive Health Care Assistance Center, Parc Sanitari Sant Joan de Deu, Cerdanyola del Vallès, Spain; ^5^ Maternal Fetal Medicine Service, Hospital Clinic, Barcelona, Spain; ^6^ Clinical Health Psychology Section, Institute of Neuroscience, Hospital Clinic, Barcelona, Spain; ^7^ Departament of Clinical and Health Psychology, Universitat Autònoma de Barcelona, Barcelona, Spain; ^8^ Institut d’Investigacions Biomèdiques August Pi i Sunyer (IDIBAPS), Barcelona, Spain

**Keywords:** EMDR recent traumatic episode protocol, stillbirth, perinatal loss, post-traumatic symptoms, PTSD, perinatal grief

## Abstract

**Background:**

Perinatal loss is a situation with significant traumatic potential. However, no study has evaluated the effectiveness of eye movement desensitization and reprocessing (EMDR) for the prevention or treatment of post-traumatic symptoms in perinatal loss. The aim of this study was to assess the feasibility and preliminary effectiveness of an EMDR recent traumatic episode protocol (EMDR-RTEP) as a preventive intervention for post-traumatic symptoms after perinatal loss.

**Materials and methods:**

A one-site, open label, randomized controlled pilot trial was designed. A total of 40 women who had suffered perinatal loss in the Maternal-Fetal Medicine Service of a tertiary university hospital were recruited. The women were randomized to the EMDR-RTEP (n=20) or treatment as usual (TAU, n=20). Post-traumatic, depressive, anxiety and intensity of perinatal grief symptoms were assessed using standardized instruments at baseline and 3 months after perinatal loss (post-treatment). We performed an intention-to-treat analysis using analysis of covariance with baseline scores as covariates.

**Results:**

Women who received the EMDR-RTEP scored non-significantly lower than women who received TAU in all post-treatment outcomes. According to a satisfaction scale (CRES-4), women who received the EMDR-RTEP had a higher perceived emotional change than women who received TAU (U = 69.5, p = .011).

**Conclusions:**

EMDR-RTE is a feasible intervention, that is well accepted and tolerated by women after perinatal loss, with adequate satisfaction. Further studies with a larger sample size are needed to confirm these results.

**Clinical Trial Registration:**

https://www.clinicaltrials.gov/, identifier NCT05701137.

## 1 Introduction

Perinatal grief has traditionally been an unacknowledged and socially unrecognized form of bereavement, that has only recently received the research attention it deserves. Grief response to perinatal loss occurs independently of the gestational week at which the loss occurs and regardless of whether the loss was the result of medical termination of pregnancy (therapeutic abortion due to severe fetal illness or malformation) or occurred unexpectedly. Additionally, perinatal loss is a potentially traumatic event that is associated with significant direct and indirect economic, psychological, social, familial, and community costs (1). Perinatal loss can have an impact on mental health (2). Between 8% and 20% of women who experience perinatal show symptoms for moderate depression (3, 4). Moreover, 20–39% of these women may develop post-traumatic stress disorder (PTSD) at one month after the loss, and approximately 4% subsequently develop chronic PTSD (5, 6). Furthermore, women with a history of fetal loss have increased anxiety and depression levels in subsequent pregnancies (7).

Limited and low-quality evidence suggests that psychosocial interventions may be effective in reducing depressive, anxious, and grief symptoms in parents after perinatal loss (8). Most evidence is based on studies evaluating the efficacy of counseling, primarily conducted by nurses or midwives (8).

There is less evidence regarding the efficacy of psychological treatments for perinatal loss, particularly in relation to improving post-traumatic symptoms (9). Kersting et al. (10) evaluated the efficacy of internet-based cognitive behavioral therapy in a sample of women after pregnancy loss (miscarriage, termination due to fetal anomaly, or stillbirth), showing a reduction in post-traumatic symptoms, grief intensity, and general psychopathology in the intervention group compared to a waiting list control group. In a subsequent randomized controlled trial by the same research team, the same intervention showed a positive effect in a larger sample of parents with pregnancy loss, with reductions in post-traumatic, depressive, anxious, and prolonged grief symptoms compared to a waiting list control group (11). Navidian et al. (12) found that four sessions of grief counseling were effective in reducing the severity of PTSD symptoms in women who had recently experienced a stillbirth. In contrast, a yoga intervention was not effective in reducing post-traumatic symptoms (13).

According to clinical practice guidelines, eye movement desensitization and reprocessing (EMDR) is a psychological therapy of choice for PTSD (14–17). Early EMDR interventions have also shown benefits in the short-term improvement of post-traumatic symptoms in populations exposed to traumatic events (18). EMDR has been applied in populations experiencing traumatic loss, offering promising results, although more studies are needed (19–21). In the perinatal population, EMDR therapy in the recent traumatic episode protocol (EMDR-RTEP) has been applied in cases of traumatic childbirth, showing efficacy as an early preventive intervention for PTSD (22). However, no study has evaluated the effectiveness of EMDR for the prevention or treatment of post-traumatic symptoms following perinatal loss. This study aimed to assess the feasibility and preliminary effectiveness of early intervention using EMDR-RTEP for the treatment and prevention of post-traumatic symptoms after perinatal loss. A secondary aim was to evaluate the preliminary effectiveness of the protocol on depressive, anxious, and intensity of perinatal grief symptoms, which were included as secondary outcomes in this pilot trial. The inclusion of these outcomes allows exploring whether EMDR-RTEP may have broader benefits beyond post-traumatic symptoms. Another secondary aim was to assess whether satisfaction with therapy would be comparable between women receiving EMDR-RTEP and those receiving treatment as usual (TAU). We hypothesized that women who experienced perinatal loss and received EMDR-RTEP would report significantly lower post-traumatic stress symptoms three months after the loss compared to those receiving TAU. Additionally, we expected that women in the EMDR-RTEP group would show lower levels of depressive and anxious symptoms, and reduced intensity of perinatal grief. We also hypothesized that satisfaction with therapy would be comparable between groups.

## 2 Materials and methods

### 2.1 Design

A pilot, single-site, open-label, randomized, controlled parallel trial was designed, following CONSORT guidelines. The study was registered *a priori* at clinicaltrials.gov (NCT05701137) and was approved by the institutional review board of the first author’s institution (HCB/2022/1197). Written informed consent was obtained from all participants. The study was conducted in accordance with the Declaration of Helsinki.

Sample size was determined *a priori*. Sample sizes between 12 to 35 per group are recommended for a pilot study with continuous outcomes (23). Using GPower, a minimum sample size of 17 subjects per group was required to assess significant differences with an effect size of one between two independent samples with an alpha error of 5% and a power of 80%. A total of 40 participants were included: 20 were randomized to the EMDR-RTEP and 20 to TAU (n=20) (allocation ratio 1:1). Participants did not receive compensation beyond the psychological care provided through their voluntary participation in the study.

### 2.2 Randomization

The random sequence was generated using a computer-generated block of random numbers (STATA v16), by a member of the research team not involved in participant selection, inclusion, or treatment. Allocation was concealed in sealed opaque envelopes, safeguarded by a research team member unrelated to other aspects of the study. Allocation was concealed until participants were enrolled and assigned to EMDR-RTEP or TAU.

### 2.3 Study population and recruitment

The study sample was recruited from the Maternal-Fetal Medicine Service of a public university hospital in Barcelona (Spain) using a consecutive sampling strategy. Women were eligible to enroll in the study if they had been attended at the hospital for recent perinatal loss (miscarriage, stillbirth, or termination of pregnancy after a positive prenatal diagnosis), were aged 18 years or over, and had requested psychological treatment. The inclusion criteria were not based on the presence of a higher intensity of symptoms. The exclusion criteria were: 1) active substance use disorder; 2) cognitive disability; and 3) language barrier. The latter two exclusion criteria are based on minimizing the difficulty or impossibility of completing the self-administered questionnaires.

All women treated at the Maternal-Fetal Medicine Service for perinatal loss routinely received a follow-up telephone call from a nurse between the first and second week after the loss. During this call, the nurse inquired about the woman’s general physical recovery and emotional state. If signs of emotional distress were identified or if the woman expressed a need for psychological care, she was offered a referral for psychological assessment and intervention. During the study recruitment period, from April 2023 to September 2024, the nurse additionally asked patients if they were interested in receiving information about the study. Women who met the inclusion criteria and wished to participate received study information from a research team member, signed the informed consent, and completed questionnaires including sociodemographic information and symptom assessment. After providing informed consent, participants were allocated to either the EMDR-RTEP or TAU. Participants and researchers were not blinded to treatment allocation. Women again completed the symptom assessment questionnaires as well as a satisfaction questionnaire 3 months after perinatal loss (post-treatment). The follow-up evaluations were completed in November 2024.

### 2.4 Treatment

Both psychological treatments were delivered by perinatal psychologists with a minimum of five years of experience in perinatal mental health care, including the treatment of perinatal grief. The EMDR intervention was administered by a single psychologist (BS) certified in EMDR and trained in the RTEP (24). Therapists were not randomized; however, all shared similar expertise in perinatal mental health.

#### 2.4.1 EMDR-RTEP

Participants in the EMDR-RTEP group received an EMDR intervention following the RTEP of Shapiro et al. (24). Given that the memory of a recent trauma is not fully consolidated, this adaptation of the standard EMDR protocol is designed for application within days after the trauma until three months post-event. The RTEP is an EMDR-based early intervention approach that targets recent trauma through structured phases ensuring emotional safety. It identifies multiple points of disturbance across the trauma continuum. Key techniques include narrative processing with bilateral stimulation, non-verbal scanning, and telescopic processing. The application of this protocol to perinatal grief and PTSD facilitates early, adaptive integration of traumatic memories.

#### 2.4.2 TAU

Participants in the TAU group received psychological treatment involving psychological grief counseling, and non-trauma-focused cognitive-behavioral techniques. This is the standard treatment provided at the hospital where the study was conducted.

### 2.5 Measures

#### 2.5.1 PTSD checklist for the Diagnostic and Statistical Manual of Mental Disorders, Fifth Edition

The PCL-5 (25) is a self-report instrument assessing post-traumatic symptoms according to DSM-5 criteria and consists of 20 Likert-type items.

#### 2.5.2 Beck Depression Inventory-II

The BDI-II (26) is a self-report instrument assessing depressive symptoms. It consists of 21 Likert-type items.

#### 2.5.3 State-Trait Anxiety Inventory

The STAI (27) is a self-report tool assessing state and trait anxiety. The state anxiety version was used, which refers to transient feelings of tension and increased central nervous system activity. It consists of 20 4-point Likert-type items.

#### 2.5.4 Perinatal Grief Scale

The PGS (28) is a self-report instrument assessing the intensity of perinatal grief. It consists of 33 Likert-type items and includes three subscales and a total score. The total score was used in this study.

#### 2.5.5 Consumer Reports Effectiveness Score

The CRES-4 (29) is a self-report instrument assessing user satisfaction with the treatment received. It includes four items evaluating three dimensions: satisfaction, problem solution, and perceived change.

### 2.6 Statistical analysis

Baseline differences in sociodemographic and clinical variables between groups were assessed using Student’s t-, Chi-square, or Fisher’s exact tests, as appropriate. Normality of the outcome variables was assessed using the Shapiro-Wilk test. Intention-to-treat analysis was conducted using the baseline-observation-carried-forward (BOCF) method for imputing missing data. Differences between intervention groups in outcomes of interest were analyzed using analysis of covariance (ANCOVA) with baseline scores as covariates. Sensitivity analysis was performed using complete case datasets. For satisfaction analysis, differences between groups were assessed using the Mann-Whitney U test. The statistical analyses were performed using SPSS v.23 and STATA v.16.

## 3 Results

### 3.1 Recruitment and baseline assessment

**Figure 1:**
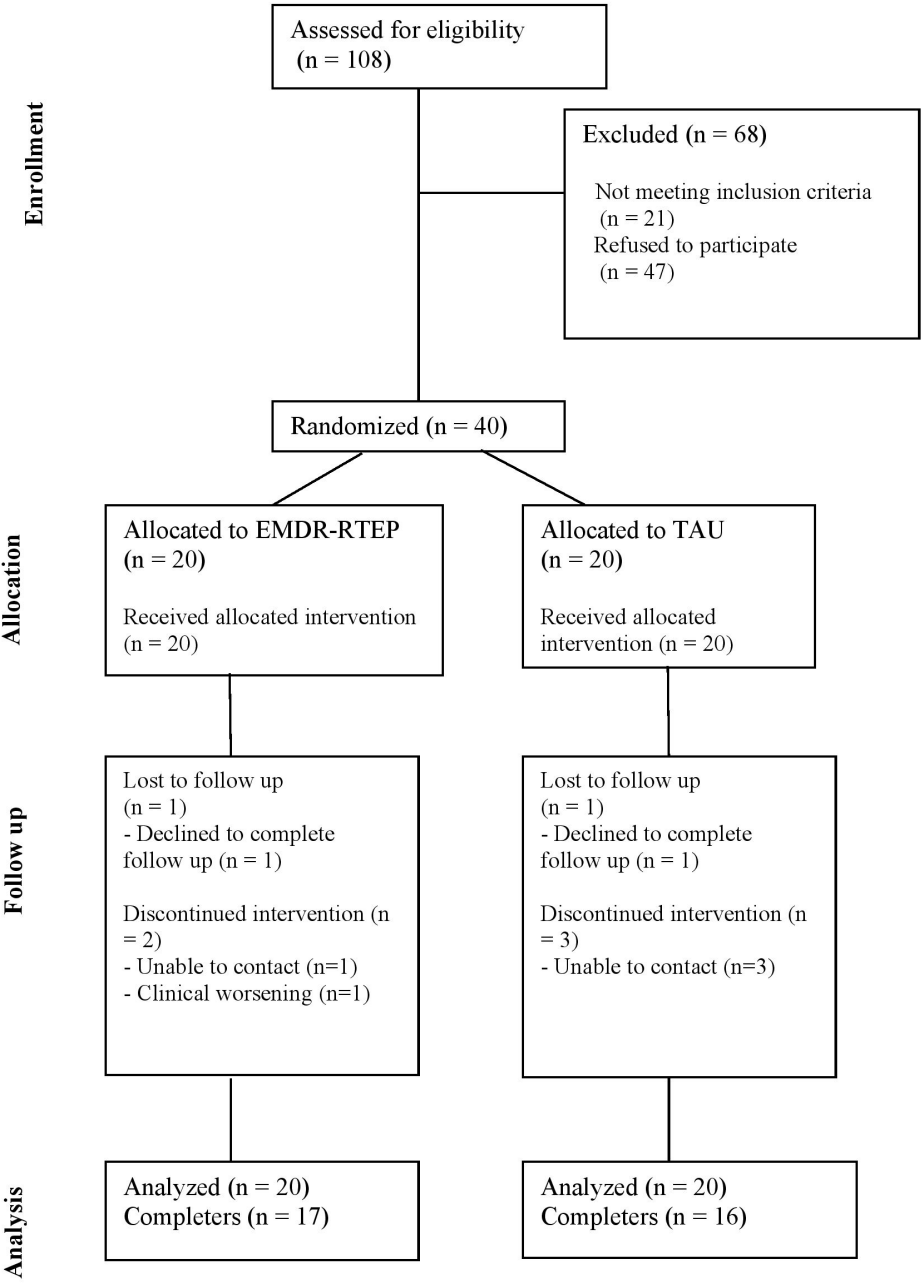
CONSORT diagram showing the flow of participants throughout the study.

Of the 108 women assessed for eligibility, 21 were excluded for not meeting the inclusion criteria, and 47 declined to participate (**Figure 1**). Consequently, 40 women were randomized, 20 allocated to EMDR-RTEP and 20 to TAU. One woman in each group completed treatment but did not respond to the post-treatment assessment, and two women in the EMDR-RTEP group and three in the TAU group discontinued the intervention. One woman in the EMDR-RTEP group abandoned the treatment due to transient clinical worsening associated with partner conflict. For the remaining women, the reason for discontinuation was unknown as contact was lost. In these cases, women reported feeling the same or better at their last visit compared to at baseline, confirming the appropriateness of the BOCF method for imputing missing data. Women in the TAU group received an average of 4.6 (standard deviation [SD] = 2.3, range = 1–10) intervention sessions, while those in the EMDR-RTEP group received an average of 5.8 (SD = 1.6, range = 3–10) sessions. The difference in the number of intervention sessions between groups was statistically significant (U = 127.0, p = .045), with women in the EMDR-RTEP group receiving, on average, one more session than those in the TAU group.

**Table 1** shows baseline assessment results by intervention group (EMDR-RTEP or TAU). No significant differences were found between the TAU and the EMDR-RTEP group in sociodemographic or clinical variables at baseline. Neither were there significant differences between the two groups in baseline scores for post-traumatic (t[38] = 0.31, p = .761), depressive (t[38] = 0.11, p = .912), anxious (t[38] = 0.46, p = .649), or perinatal grief (t[38] = 0.56, p = .582) symptoms. The mean scores of the questionnaires at baseline and post-treatment are shown in **Table 2**.

**Table 1:** Baseline characteristics.

Variables	TAU (N = 20)	EMDR-RTEP (N = 20)	*p- value*
Nationality – N (%)			.731
Spanish Foreigner	13 (65%) 7 (35%)	15 (75%) 5 (25%)	
Education – N (%)			.686
No education Primary education Secondary education University education	1 (5%) 3 (15%) 6 (30%) 10 (50%)	0 (0%) 2 (10%) 8 (40%) 10 (50%)	
Age – mean (SD)	35.4 (3.7)	35.3 (6.0)	.950
Live children – N (%)			.341
Yes No	11 (55%) 9 (45%)	7 (35%) 13 (65%)	
Employment – N (%)			.605
Yes No	17 (85%) 3 (15%)	19 (95%) 1 (5%)	
Financial problems – N (%)			.661
Yes No	4 (20%) 16 (80%)	2 (10%) 18 (90%)	
Partner – N (%)			1
Yes No	18 (90%) 2 (10%)	19 (95%) 1 (5%)	
Partner relationship – N (%)			.744
No partner Close and warm Sporadic disagreements	2 (10%) 9 (45%) 9 (45%)	1 (5%) 11 (55%) 8 (40%)	
Previous perinatal loss – N (%)			.273
Yes No	3 (15%) 17 (85%)	7 (35%) 13 (65%)	
Assisted reproduction – N (%)			.182
Yes No	1 (5%) 19 (95%)	5 (25%) 15 (75%)	
Type of perinatal loss – N (%)			.127
TOP Stillbirth	13 (65%) 7 (35%)	18 (95%) 2 (5%)	
Gestational age – mean (SD)	24.9 (5.5)	24.7 (4.7)	.876
Pregnancy planning – N (%)			.235
Planned Unplanned	14 (70%) 6 (30%)	18 (90%) 2 (10%)	
Psychiatric history – N (%)			.514
Yes No	6 (30%) 14 (70%)	9 (45%) 11 (55%)	
Family psychiatric history – N (%)			.748
Yes No	7 (35%) 13 (65%)	9 (45%) 11 (55%)	

EMDR-RTEP, Eye movement desensitization and reprocessing – recent traumatic episode protocol; TAU, treatment as usual; TOP, termination of pregnancy; SD, standard deviation.

**Table 2:** Mean scores on outcome measures in each group at baseline and post-treatment.

Measure	Assessment	TAU			EMDR-RTEP		
		N	M	SD	N	M	SD
PCL-5	Baseline	20	36.5	16.6	20	34.8	17.4
	Post-treatment	16	28.3	17.5	17	24.2	15.5
BDI-II	Baseline	20	25.1	12.3	20	24.6	13.4
	Post-treatment	16	19.3	12.5	17	15.2	7.1
STAI-S	Baseline	20	36.7	11.7	20	34.9	13.1
	Post-treatment	16	29.3	12.2	17	25.2	10.1
PGS	Baseline	20	105.2	23.7	20	101.4	17.1
	Post-treatment	16	98.5	27.0	17	89.2	19.5

EMDR-RTEP, Eye movement desensitization and reprocessing – Recent traumatic episode protocol; TAU, treatment as usual; PCL-5, PTSD Checklist for DSM-5; BDI-II, Beck Depression Inventory – II; STAI-S, State Trait Anxiety Inventory – State; PGS, Perinatal Grief Scale; M, mean; N, number; SD, standard deviation.

### 3.2 Intervention outcomes

**Figure 2:**
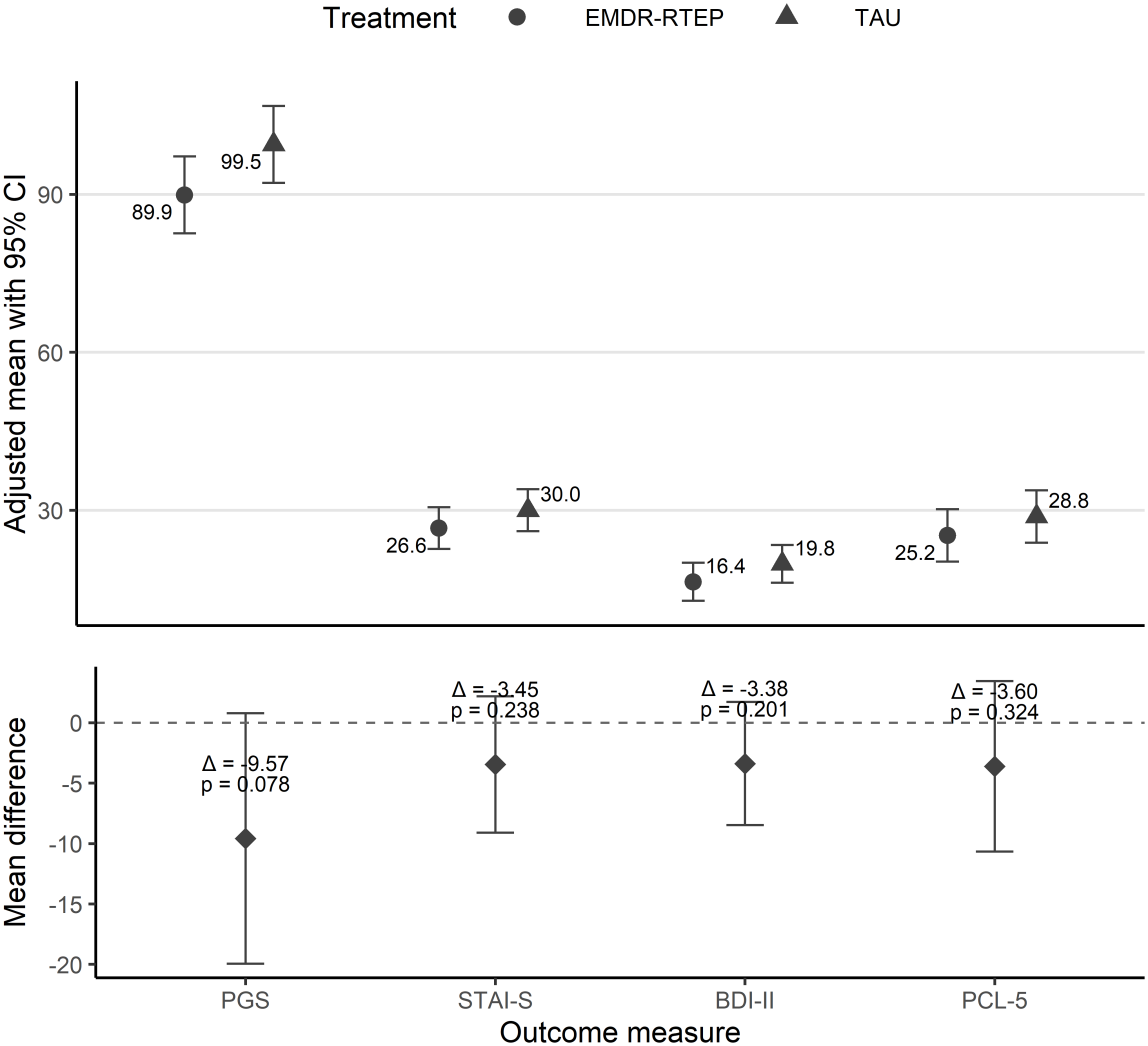
Differences between EMDR-RTEP and TAU on outcome scores post-treatment in the intention-to-treat analysis. Estimated marginal means from ANCOVA models adjusted for baseline scores. Adjusted mean differences between EMDR-RTEP and TAU. Negative values favor EMDR-RTEP, while positive values favor TAU. 0 = no differences. EMDR-RTEP: Eye Movement Desensitization and Reprocessing – Recent traumatic episode; TAU: treatment-as-usual; PCL-5: PTSD Checklist for DSM-5; BDI-II: Beck Depression Inventory – II; STAI-S: State Trait Anxiety Inventory – State; PGS: Perinatal Grief Scale.

The ANCOVA models with imputed data showed no statistically significant differences between the two intervention groups (EMDR-RTEP and TAU) in post-traumatic (F[1,37] = 0.99, p = .324, ηp² = .03), depressive (F[1,37] = 1.69, p = .201, ηp² = .04), anxious (F[1,37] = 1.44, p = .238, ηp² = .04), or perinatal grief (F[1,37] = 3.28, p = .078, ηp² = .08) symptoms. **Figure 2** shows the estimated marginal means from the ANCOVA models and adjusted mean differences between the EMDR-RTEP and TAU groups, with lower scores in all intervention outcomes in the EMDR-RTEP group compared to TAU, although these differences were not statistically significant. Sensitivity analysis using complete case data yielded results consistent with the primary analysis (Supplementary Material, **Supplementary Figure 1**).

### 3.3 Satisfaction with the treatment received

**Figure 3:**
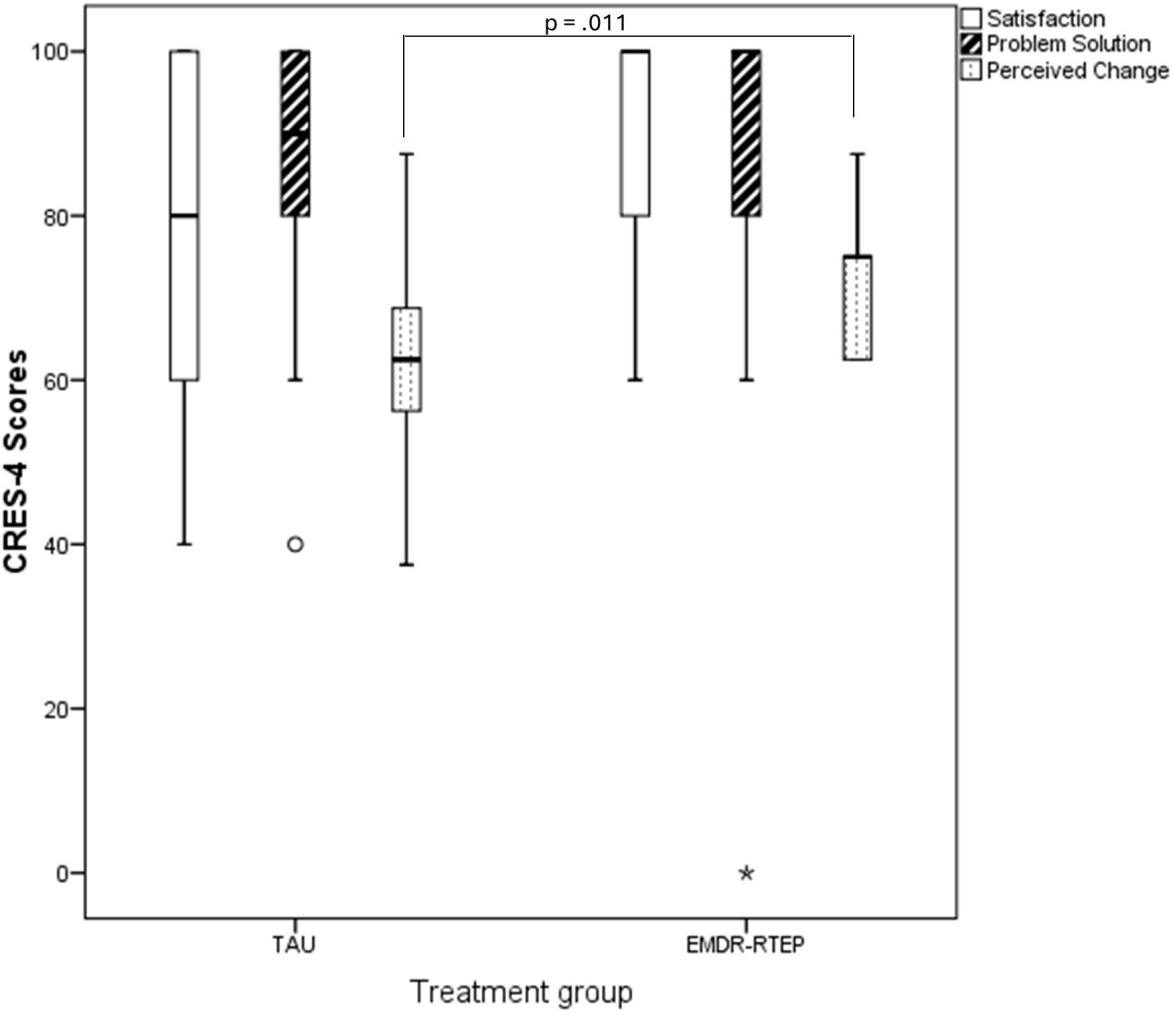
Distribution of satisfaction scale scores (CRES-4) by group. EMDR-RTEP, Eye Movement Desensitization and Reprocessing – Recent traumatic episode protocol; TAU, treatment-as-usual; CRES-4, Consumer Reports Effectiveness Score.

**Figure 3** shows the distribution of the scores of the satisfaction scale (CRES-4). Non-parametric mean difference tests showed no significant differences between groups in relation to satisfaction (U = 90, p = .071) or problem solution (U = 125.5, p = .673). However, patients who received the EMDR-RTEP reported a higher perceived change than those who received TAU (U = 69.5, p = .011).

## 4 Discussion

To our knowledge, this is the first study to evaluate and compare the feasibility and preliminary effectiveness of an early EMDR intervention with a TAU intervention control group for the treatment and prevention of post-traumatic symptoms following perinatal loss. The control group received psychological grief counseling, and non-trauma-focused cognitive-behavioral techniques.

Both groups showed a trend toward decreased scores post-treatment compared to baseline. At post-treatment assessment, the EMDR-RTEP group had lower scores in all outcomes compared to the TAU group, although these differences were not statistically significant. Both treatments were well-tolerated, with no significant adverse effects directly related to the interventions. Mothers were generally satisfied with the treatment received, although those who received the EMDR-RTEP reported greater perceived emotional change compared to those who received TAU, being the only result achieving statistical significance.

The goal of a pilot study is to evaluate the feasibility of an intervention and estimate the potential effect size to help in the design of a larger definitive trial (30). This justifies the use of less conservative confidence intervals (CI) (e.g., 85% or 75%) along with the minimum clinically important difference (MCID) (30). The MCID is typically set at 3 for the BDI-II (31), while recent studies suggest 9 for the PCL-5 (32) and 10 for the STAI-S (33, 34). Considering the CIs for mean differences between the EMDR-RTEP and TAU, the 75% CI for BDI-II scores (0.3–6.4) excludes 0 and includes the MCID. The 85% and 75% CI for PGS scores ([1.8–17.3] and [3.4–15.7], respectively) both exclude 0. Although the MCID for this scale is unknown, the differences are likely to be clinically significant. This corresponds to a medium effect size (ηp² = .08). Additionally, women who received the EMDR-RTEP reported greater perceived emotional change than those who received TAU. These findings suggest that early EMDR intervention may be more beneficial than psychological interventions involving grief counseling, and non-trauma-focused cognitive-behavioral techniques for women experiencing perinatal loss. Surprisingly, the trend towards improvement seemed less evident in post-traumatic symptoms and greater in secondary outcomes, such as depressive symptoms and the intensity of perinatal grief. This hypothesis should be tested in a larger clinical trial.

Compared to previous studies on psychological therapy, prior trials have shown that cognitive-behavioral therapy (10, 11) yields beneficial results in preventing or treating post-traumatic symptoms in perinatal grief. However, in contrast to our study, the comparison group did not receive an active psychological intervention.

From a clinical perspective, this pilot study highlights the importance of integrating trauma-focused treatments into perinatal mental health care, particularly for interventions in women who have suffered a recent perinatal loss. The study also supports the acceptability of EMDR-RTEP in this context, with high levels of treatment adherence and satisfaction among participants. In terms of research implications, these preliminary results provide a strong rationale for conducting a larger, multicenter, randomized controlled trial to assess the efficacy of EMDR-RTEP, exploring long-term outcomes, and examining the effects of this protocol on other domains, such as perinatal grief intensity and comorbid symptoms. Future studies should also consider incorporating qualitative methodologies to capture the subjective experiences of patients.

This randomized controlled trial has several strengths. Firstly, the use of a control group (TAU) allowed direct comparison of the effects of the EMDR-RTEP intervention with those of standard psychological treatment, including psychoeducation and cognitive behavioral techniques. Both interventions were delivered by psychologists with expertise in perinatal mental health. However, this pilot trial also has several limitations. Firstly, the sample size was small, and the study was conducted at a single center, which may limit the generalizability of the findings. A definitive trial with a larger sample size and multicenter design is required to increase external validity and control for therapist-related effects. Secondly, while both groups received structured psychological interventions, the EMDR-RTEP intervention was delivered by one therapist, whereas the TAU sessions were delivered by other psychologists. The therapists were not randomized and, although they all had comparable clinical experience in perinatal mental health, differences in their therapeutic styles and rapport may have influenced the outcomes. Thirdly, the number of sessions received differed significantly between the two groups: on average, the EMDR-RTEP group received one more session than the TAU group. This difference may have contributed to the effects observed. Fourthly, symptom improvement was assessed using standardized self-reported scales administered before and after the intervention, which may not fully capture the therapeutic process. Additionally, we used a brief satisfaction scale to assess participants’ perceptions of the intervention, which may be insufficient for evaluating treatment impact. Future studies would benefit from incorporating qualitative methodologies to explore patients’ experiences and perceived changes in greater depth. Lastly, for ethical reasons, the control group in our study received active psychological treatment, and thus, we lack a comparison between the EMDR-RTEP and no treatment or a waiting list.

## 5 Conclusion

It is important to expand the evidence base for psychological treatments for perinatal grief, particularly for post-traumatic symptoms following perinatal loss. This pilot study shows that the intervention is feasible, well accepted, and tolerated by participants, with adequate satisfaction levels. It requires a slightly higher number of psychological visits compared to routine treatment and involves specific training. Although there was no clear trend towards improvement in post-traumatic symptoms, the intervention yielded promising results on secondary outcomes, particularly in reducing depressive symptoms and the intensity of perinatal grief. These findings warrant further investigation in larger, controlled trials.

## Data Availability

The raw data supporting the conclusions of this article will be made available by the authors, without undue reservation.
