# Supplementary material for: Effectiveness of an eye movement desensitization and reprocessing intervention for the prevention of post- traumatic symptoms in perinatal loss: a randomized pilot controlled trial
Source: Front Psychiatry. 2025 Jun 9;16:1593306. doi: 10.3389/fpsyt.2025.1593306 (PMC12183222; doi:10.3389/fpsyt.2025.1593306)
Supplement: Supplementary file 1 [file DataSheet1.pdf]

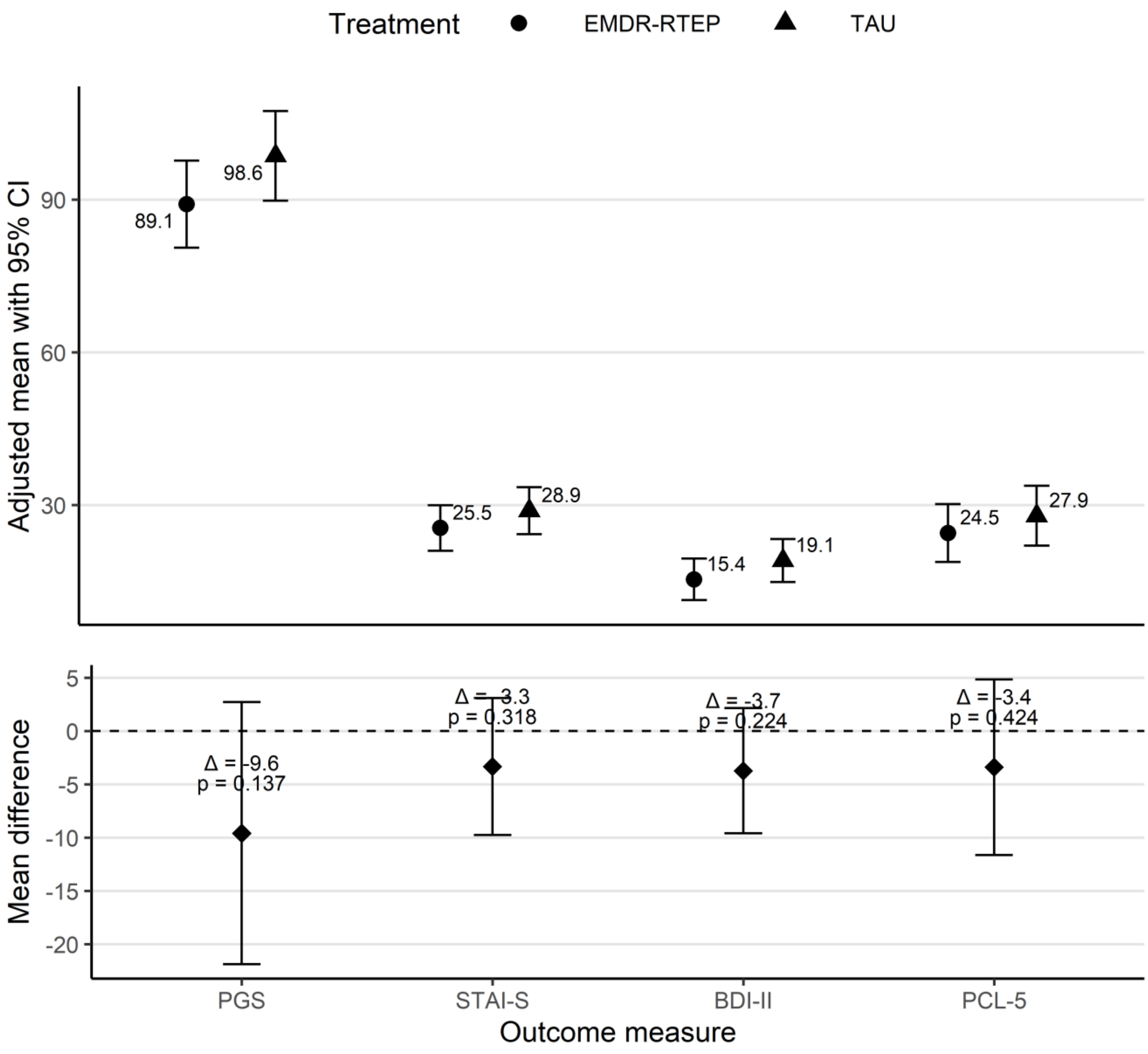

Supplementary Figure 1. Differences between EMDR-RTEP and TAU on outcome scores in complete case dataset. Estimated marginal means from ANCOVA models adjusted for baseline scores. Adjusted mean differences between EMDR-RTEP and TAU. Negative values favour EMDR-RTEP, while positive values favour TAU. 0 = no differences. EMDR-RTEP: Eye Movement Desensitization and Reprocessing – Recent traumatic episode; TAU: treatment-as-usual; PCL-5: PTSD Checklist for DSM-5; BDI-II: Beck Depression Inventory – II; STAI-S: State Trait Anxiety Inventory – State; PGS: Perinatal Grief Scale.
